# The pyruvate decarboxylase activity of IpdC is a limitation for isobutanol production by *Klebsiella pneumoniae*

**DOI:** 10.1186/s13068-022-02144-8

**Published:** 2022-05-02

**Authors:** Lin Shu, Jinjie Gu, Qinghui Wang, Shaoqi Sun, Youtian Cui, Jason Fell, Wai Shun Mak, Justin B. Siegel, Jiping Shi, Gary J. Lye, Frank Baganz, Jian Hao

**Affiliations:** 1 Lab of Biorefinery, Shanghai Advanced Research Institute, Chinese Academy of Sciences, No. 99 Haike Road, Pudong, Shanghai, 201210 People’s Republic of China; 2 Department of Biochemical Engineering, University College London, Gordon Street, London, WC1H 0AH UK; 3 Department of Chemistry, Biochemistry & Molecular Medicine, and the Genome Center, University of California, Davis, One Shields Avenue, Davis, CA 95616 USA; 4 University of Chinese Academy of Sciences, Beijing, 100049 People’s Republic of China

**Keywords:** Isobutanol, 2-Ketoisovalerate decarboxylase, Indole-3-pyruvate decarboxylase, *Klebsiella pneumoniae*

## Abstract

**Background:**

*Klebsiella pneumoniae* contains an endogenous isobutanol synthesis pathway. The *ipdC* gene annotated as an indole-3-pyruvate decarboxylase (Kp-IpdC), was identified to catalyze the formation of isobutyraldehyde from 2-ketoisovalerate.

**Results:**

Compared with 2-ketoisovalerate decarboxylase from *Lactococcus lactis* (KivD), a decarboxylase commonly used in artificial isobutanol synthesis pathways, Kp-IpdC has an 2.8-fold lower *K*_m_ for 2-ketoisovalerate, leading to higher isobutanol production without induction. However, expression of *ipdC* by IPTG induction resulted in a low isobutanol titer. In vitro enzymatic reactions showed that Kp-IpdC exhibits promiscuous pyruvate decarboxylase activity, which adversely consume the available pyruvate precursor for isobutanol synthesis. To address this, we have engineered Kp-IpdC to reduce pyruvate decarboxylase activity. From computational modeling, we identified 10 amino acid residues surrounding the active site for mutagenesis. Ten designs consisting of eight single-point mutants and two double-point mutants were selected for exploration. Mutants L546W and T290L that showed only 5.1% and 22.1% of catalytic efficiency on pyruvate compared to Kp-IpdC, were then expressed in *K. pneumoniae* for in vivo testing. Isobutanol production by *K. pneumoniae* T290L was 25% higher than that of the control strain, and a final titer of 5.5 g/L isobutanol was obtained with a substrate conversion ratio of 0.16 mol/mol glucose.

**Conclusions:**

This research provides a new way to improve the efficiency of the biological route of isobutanol production.

**Supplementary Information:**

The online version contains supplementary material available at 10.1186/s13068-022-02144-8.

## Highlights

1. Kp-IpdC is more efficient than KivD for 2-ketoisovalerate decarboxylation.
2. Pyruvate decarboxylase activity is a limitation of Kp-IpdC.
3. T290L variant exhibits a decreased pyruvate decarboxylase activity.
4. Isobutanol production by *K. pneumoniae* T290L was improved.

**Supplementary Information:**

The online version contains supplementary material available at 10.1186/s13068-022-02144-8.

## Background

Production of sustainable bioenergy for industrial usage is a very important capability of biotechnology. This technology tries to find solutions for today’s globally significant ecological and energy challenges [1]. The primary biofuel for the gasoline market has historically been ethanol manufactured from corn. However, a number of drawbacks have been identified: it has a lower energy content compared to gasoline, it is not amenable to pipeline distribution, and the amount that can be blended into gasoline is limited [2]. By contrast, higher molecular weight alcohols such as isobutanol and *n*-butanol have higher energy contents and should be more amenable to pipeline distribution [3]. Isobutanol can be blended with diesel and biodiesel in high ratios [4]. Compared to diesel fuel, CO and NOx emissions decrease with the use of blends of isobutanol and diesel [5]. Thus, isobutanol is considered to be a new generation of biofuel.

*Saccharomyces cerevisiae* was previously considered as the only known natural microorganism that can synthesize isobutanol at a detectable level. In addition, isobutanol and other higher molecular weight alcohols are by-products of the bioethanol industry [6]. These higher molecular weight alcohols are synthesized from amino acids via an Ehrlich pathway. Isobutanol can be also de novo synthesized from pyruvate. First, pyruvate is imported into the mitochondria and two molecules are condensed to acetolactate by acetolactate synthase. In the subsequent step, acetolactate is reduced to 2,3-dihydroxyisovalerate by acetohydroxyacid reductoisomerase. Finally, 2,3-dihydroxyisovalerate is converted to 2-ketoisovalerate by dihydroxyacid dehydratase. 2-ketoisovalerate is then transported into the cytosol to form isobutyraldehyde and further reduced to isobutanol [7]. Many metabolic engineering works have been done, and isobutanol synthesized by *S. cerevisiae* have been improved to 2.06 g/L [8]. However, the main metabolite produced by *S. cerevisiae* is still ethanol.

With the development of synthetic biology, an artificial isobutanol synthesis pathway was established in *Escherichia coli* [9]. This pathway was similar to that of *S. cerevisiae* using α-acetolactate synthase from *Bacillus subtilis*, acetohydroxyacid reductoisomerase and dihydroxyacid dehydratase from *E. coli*, 2-ketoisovalerate decarboxylase and alcohol dehydrogenase from *Lactococcus lactis* to catalyze all reactions in the synthesis pathway [10]. Following this strategy, the isobutanol synthesis pathway was constructed in many different microorganisms, and *E. coli* produced the highest isobutanol level so far with the titer of 56.5 g/L [11].

**Fig. 1:**
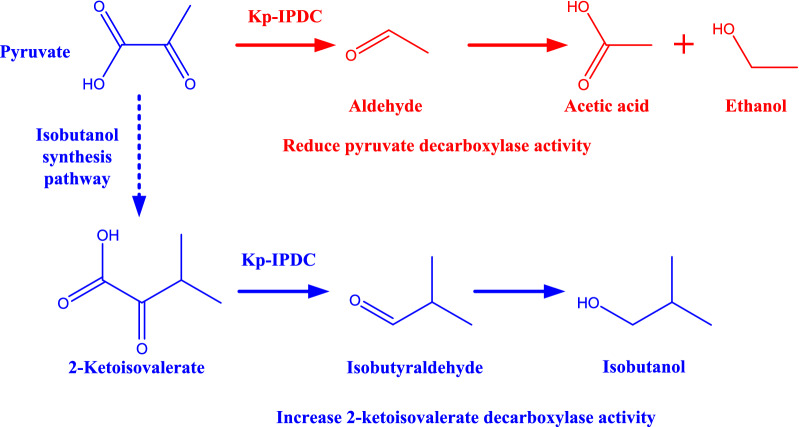
Metabolic pathway of isobutanol synthesis and branch pathway in *K. pneumoniae*. Kp-IpdC is catalysing the 2-ketoisovalerate decarboxylation and further conversion to isobutanol (blue route). It was suspected that the pyruvate decarboxylase activity of Kp-IpdC reduced the available pyruvate for isobutanol synthesis (red route)

*Klebsiella pneumoniae* is an important industrial microorganism, it has the advantages of growing at a fast rate and has a low contamination risk by other bacteria. Wild-type *K. pneumoniae* is an efficient 1,3-propanediol and 2,3-butanediol producer [12, 13]. Engineered strains of *K. pneumoniae* have been constructed for many chemicals production [14–17]. A acetolactate decarboxylase (*budA*) disrupted strain of *K. pneumoniae* was found to synthesize isobutanol via an endogenous pathway [18]. This endogenous isobutanol synthesis pathway consisted of the same steps as the artificial isobutanol synthesis pathway that was constructed in *E. coli*. An indole-3-pyruvate decarboxylase (Kp-IpdC) that is encoded by *ipdC* was identified to catalyze the isobutyraldehyde formation from 2-ketoisovalerate in *K. pneumoniae* (Fig. 1). A considerable level of 2-ketoisovalerate was accumulated in the broth, which indicated the 2-ketoisovalerate decarboxylation was a bottleneck of this isobutanol synthesis pathway. To improve isobutanol production, *ipdC* was overexpressed on a plasmid. However, expression of *ipdC* with IPTG induction resulted in a decrease of isobutanol production [18].

The objective of the present study was to reveal the mechanism of Kp-IpdC in catalysis of 2-ketoisovalerate decarboxylation and to improve its catalytic performance for isobutanol production.

## Material and methods

### Strains, plasmids, and primers

Bacterial strains and plasmids used in this study are listed in Table 1. Primers used for PCR are listed in Additional file 1: Table S1. *K. pneumoniae* CGMCC 1.6366 is an industrial strain that is used for 1,3-propanediol and other chemicals production. Chemicals used in this study were purchase from Sinopharm Chemical Reagent Co., Ltd.

**Table 1:** Strains and plasmids

Strains or plasmids	Relevant genotype and description	References
*K. pneumoniae* strains		
CGMCC 1.6366	TUAC01 Wild type	[12]
Δ*budA*–Δ*ldhA*–*ipdC*	Δ*budA*, Δ*ldhA*, pDK6*-ipdC*	This work
Δ*budA*–Δ*ldhA*–*kivD*	Δ*budA*, Δ*ldhA*, pDK6*-kivD*	This work
IpdC	Δ*budA*, Δ*ldhA*, Δ*ipdC*, pDK6*-ipdC*	[18]
KivD	Δ*budA*, Δ*ldhA*, Δ*ipdC*, pDK6*-kivD*	This work
T290L	Δ*budA*, Δ*ldhA*, Δ*ipdC*, pDK6*-ipdC-T290L*	This work
L546W	Δ*budA*, Δ*ldhA*, Δ*ipdC*, pDK6*-ipdC-L546W*	This work
*E. coli* strains		
*E. coli DH5α*	Host of plasmid	Lab stock
BL21(DE3)	Host of plasmid	Lab stock
BL21/*ipdC*	Carries pET28a-*ipdC*	This work
BL21/*kivD*	Carries pET28a-*kivD*	This work
BL21/D289L	Carries pET28a-*ipdC-*D289L	This work
BL21/T290L	Carries pET28a-*ipdC-*T290L	This work
BL21/Q383M	Carries pET28a-*ipdC-*Q383M	This work
BL21/A387I	Carries pET28a-*ipdC-*A387I	This work
BL21/F388W	Carries pET28a-*ipdC-*F388W	This work
BL21/A387L	Carries pET28a-*ipdC-*A387L	This work
BL21/V542I	Carries pET28a-*ipdC-*V542I	This work
BL21/L546W	Carries pET28a-*ipdC-*L546W	This work
BL21/D289L + T290L	Carries pET28a-*ipdC-*D289L + T290L	This work
BL21/A387I + F388W	Carries pET28a-*ipdC-*A387I + F388W	This work
Plasmids		
pDK6	Kan^r^, lacIQ, tac, 5.1 kb	[19]
pDK6-*kivD*	pDK6 carries *kivD*	[18]
pDK6-*ipdC*	pDK6 carries *ipdC*	[18]
pET28a	Vector carries N-terminal His Tag, Kan^r^, 5369 bp	Novagen^®^
pGEM-*kivD*	Vector holding *kivD* (*L. lactis*)	[20]
pMD18-T-*ipdC*	Amp^r^, carries *ipdC*	[18]
pET28a-*kivD*	pET28a carries *kivD*	This work
pET28a-*ipdC*	pET28a carries *ipdC*	This work

### Strains and plasmids construction

*Escherichia coli* BL21 was used as host for Kp-IpdC heterologous expression. pMD18-T-*ipdC* was a cloning vector that holds *ipdC*. This plasmid was digested with *EcoRI* and *BamHI* to obtain the *ipdC* fragment, and this fragment was ligated into pET28a to generate pET28a-*ipdC*. pET28a-*ipdC* was transformed into *E. coli* BL21 for protein expression. BL21*/kivD* was constructed using the same approach as BL21/*ipdC*.

Oligonucleotide-directed site-specific mutagenesis was carried out on expression plasmids of Kp-IpdC variants. pET28a-L546W was constructed based on pET28a-*ipdC*. Primer pair L546W-s and L546W-a were used to amplify pET28a-L546W with pET28a-*ipdC* as the template. The PCR product was transformed into *E. coli* BL21 to obtain BL21/L546W. Other mutants of *ipdC* expression strains were constructed using the same approach.

pDK6 was a vector used for gene expression in *K. pneumoniae* strains. pDK6-L546W was constructed in the same way as pET28a-L546W with pDK6-*ipdC* replacing pET28a-*ipdC* as the template. pDK6-L546W was transformed into *K. pneumoniae* Δ*budA*–Δ*ldhA*–Δ*ipdC* to obtain *K. pneumoniae* L546W.

### Enzymatic reaction kinetic parameters determination

BL21/*ipdC*, BL21/*kivD*, and other *E. coli* strains expressing mutants of *ipdC* were cultured in Luria–Bertani (LB) medium with 1 mM IPTG added after 4 h of cultivation at 37 °C. After culturing overnight, cells were collected from 50 mL broth by centrifugation. Pelleted cells were washed twice and then resuspended for cell lysate preparation. Sonication was performed in a tube immersed in ice-water with a pulse duration of 3 s on 3 s off, for a total of 99 cycles. Cell debris were removed from the cell lysate by centrifugation and purified enzyme was obtained through a His tag Ni–NTA–Sefinose Column (Sangon Biotech, Shanghai, China) by following the protocol given by the manufacturer.

Enzyme activities of KivD, KP-IpdC, and variants of Kp-IpdC were determined by a coupled enzymatic method. The method was based on the ability of alcohol dehydrogenase, in the presence of NADH, to reduce aldehydes formed from 2-keto acid by decarboxylase. The reaction was measured spectrophotometrically by the decrease in optical density at 340 nm using a NanoDrop 2000c spectrophotometer (Thermo Fisher Scientific, USA). Pyruvate and 2-ketoisovalerate were used as substrates individually. The reaction mixture contained 50 mM potassium phosphate, 1 mM MgSO_4_·7H_2_O, 0.5 mM thiamine pyrophosphate, 0.2 mM NADH, 45 U/mL alcohol dehydrogenase of *S. cerevisiae* (Sangon Biotech, Shanghai, China). The reaction was initiated by adding the substrates. Kinetic data were fitted to the Lineweaver–Burk plot, and the parameters such as *K*_m_, *V*_max_, and *K*_cat_ of enzymes were determined from a linear least-squares fit (OriginPro 2018, OriginLab Corp., USA).

### Medium and culture condition

The fermentation medium contained 100 g/L glucose, 5 g/L yeast extract, 4 g/L corn steep liquor, 5 g/L (NH_4_)_2_SO_4_, 3 g/L sodium acetate, 0.4 g/L KCl and 0.1 g/L MgSO_4_. For the seed culture, 250-mL flasks containing 50 mL of LB medium were incubated on a rotary shaker at 37 °C and 200 rpm for 12 h. The seed culture was inoculated into a 5-L bioreactor (BIOSTAT-A plus Sartorius, Germany) with a working volume of 3 L. The culture pH was automatically controlled at 7. The air flow rate and agitation were set at 2 L/min and 300 rpm, respectively. The off-gas was fed through a glass condenser, which was immerged in an ice-bath, and the condensate was collected. All experiments were performed in triplicate, and data are expressed as the mean ± standard error.

### Analytical methods

The biomass concentration was evaluated by determination of optical density (OD 600) with a spectrophotometer (721N INESA, China).

Chemical compounds in the broth were quantified by a Shimadzu 20AVP high performance liquid chromatography system (HPLC) (Shimadzu Corp., Kyoto, Japan) equipped with a RID-10A refractive index detector and a SPD-M20A photodiode array detector. An Aminex HPX-87H column (300 × 7.8 mm) (Bio-Rad, USA) was used and the column temperature was set up at 65 °C. The mobile phase was 0.005 mol/L H_2_SO_4_ solution at a flow rate of 0.8 mL/min.

### Homology modeling of Kp-IpdC

The Rosetta software suite is an academically developed framework for protein structure prediction and design [21]. The three-dimensional structure of Kp-IpdC was modeled with RosettaCM [22, 23]. From the NCBI database 10 homologs with ≥ 30% sequence identity to Kp-IpdC were selected as templates to predict the structure. 3D structures of these homologous proteins have been solved by X-ray diffraction of its crystal structures. PDB ID of these proteins are: 1OVM, 2VBF, 1QPB, 2W93, 2VK8, 2VJY, 1PVD, 1PYD, 2VK1, and 5NPU. A total of 10,000 structure simulations were run and the structure with the lowest Rosetta energy was chosen.

### Computational Kp-IpdC redesign

RosettaDock was used to dock the substrate to Kp-IpdC and the variants of Kp-IpdC [24]. Previous reports about decarboxylase design [25] identified 10 residues (D289, T290, Q383, A387, F388, G408, V467, I471, V542, and L546) within 8 Å of the active site of Kp-IpdC for mutagenesis. Each of these 10 residues was substituted with one of 12 hydrophobic amino acids (V L I M F H G A T Y W S). The identities of amino acids at all other residues were kept constant. These variants were docked with either 2-ketoisovalerate or pyruvate as the substrate. A total of 10,000 design simulations were run, from which the 10 designs that had the most favourable Rosetta interface energy with 2-ketoisovalerate as substrate, while unfavourable Rosetta interface energy with pyruvate as substrate was selected to construct variants of Kp-IpdC.

## Results

### Isobutanol production by *K. pneumoniae* using Kp-IpdC or KivD as the decarboxylase

**Fig. 2:**
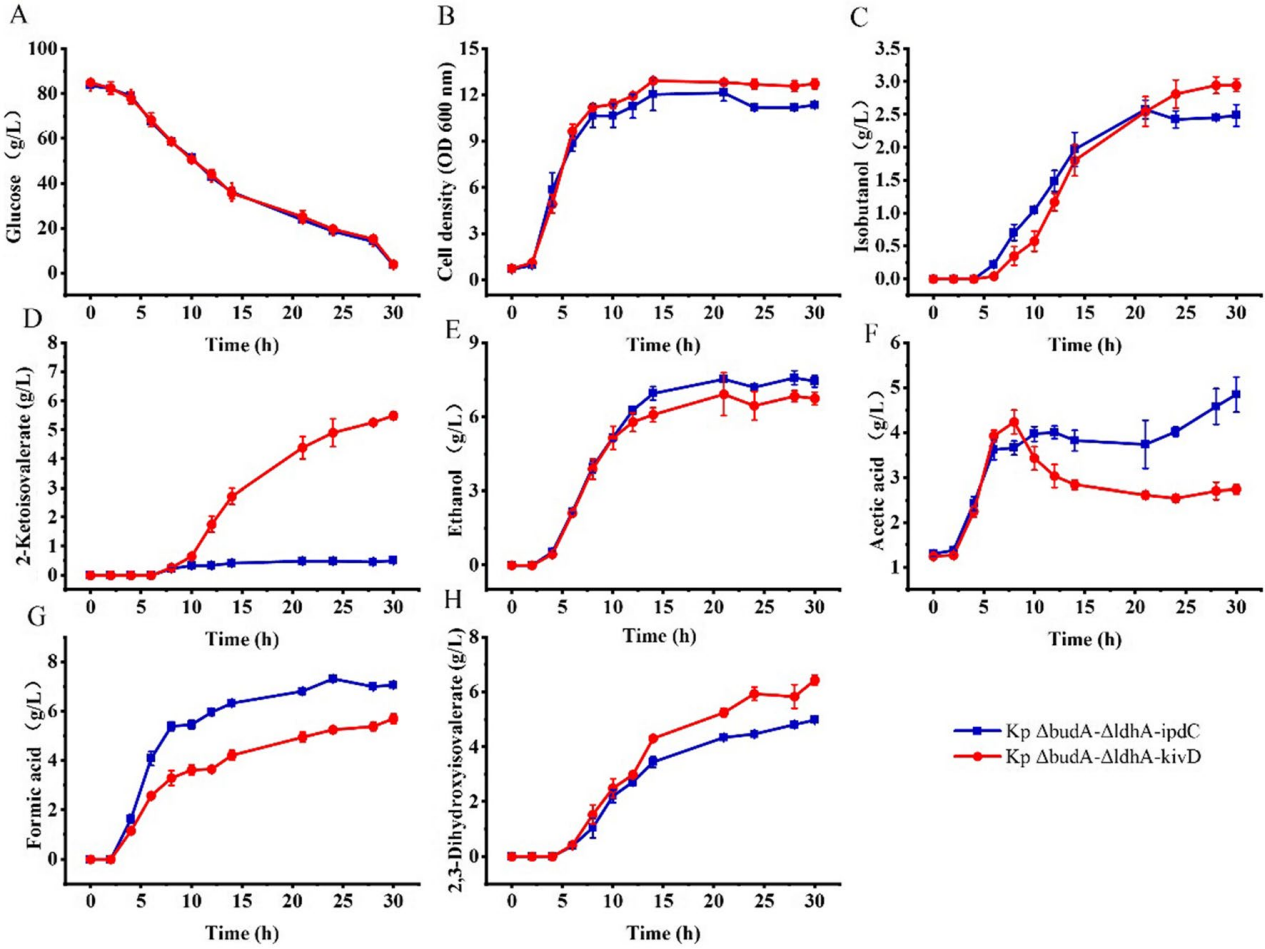
The cell growth and metabolite production of *K. pneumoniae* Δ*budA*–Δ*ldhA*–*ipdC* and *K. pneumoniae* Δ*budA*–Δ*ldhA*–*kivD* in batch culture with IPTG induction. Cells were cultured in 5 L bioreactors and 1 mM of IPTG was added to the culture broth after 8 h of cultivation. Data points are the average of *n* = 3; error bars represent standard error about the mean

*Klebsiella pneumoniae* Δ*budA*–Δ*ldhA* is an isobutanol production strain constructed previously [18]. Kp-IpdC has been identified to catalyze the reaction of isobutyraldehyde formation from 2-ketoisovalerate. KivD is an *L. lactis* decarboxylase and has been used in all artificial isobutanol synthesis pathways. *K. pneumoniae* Δ*budA*–Δ*ldhA*–*ipdC* and *K. pneumoniae* Δ*budA*–Δ*ldhA*–*kivD* were constructed to compare the difference of the two decarboxylases on isobutanol production by *K. pneumoniae*. These two strains were batch cultured in 5 L bioreactors and induced with 1 mM IPTG. The fermentation results are shown in Fig. 2.

Cell growth and glucose consumption of the two strains were comparable. Cells grew quickly in the first 10 h of cultivation and cell densities were kept stable in the remaining cultivation time. About 80 g/L of glucose was completely exhausted by both strains after 30 h of cultivation. Isobutanol produced by *K. pneumoniae* Δ*budA*–Δ*ldhA*–*ipdC* and *K. pneumoniae* Δ*budA*–Δ*ldhA*–*kivD* were 2.5 and 2.9 g/L, respectively. 2-ketoisovalerate was found to be accumulated in the broth of the two strains with titers of 0.5 and 5.5 g/L, respectively. The final titer of ethanol generated by the two strains were 7.4 and 6.8 g/L, respectively. In addition, 4.0 and 3.4 g/L of acetic acid were produced by the two strains after 10 h of cultivation. The acetic acid level decreased to 2.7 g/L for *K. pneumoniae* Δ*budA*–Δ*ldhA*–*kivD* but its final level was 4.9 g/L for *K. pneumoniae* Δ*budA*–Δ*ldhA*–*ipdC*. Formate produced by the two strains were 7.1 and 5.7 g/L, respectively. 2,3-Dihydroxyisovalerate accumulated to levels of 5.0 and 6.4 g/L, respectively.

**Fig. 3:**
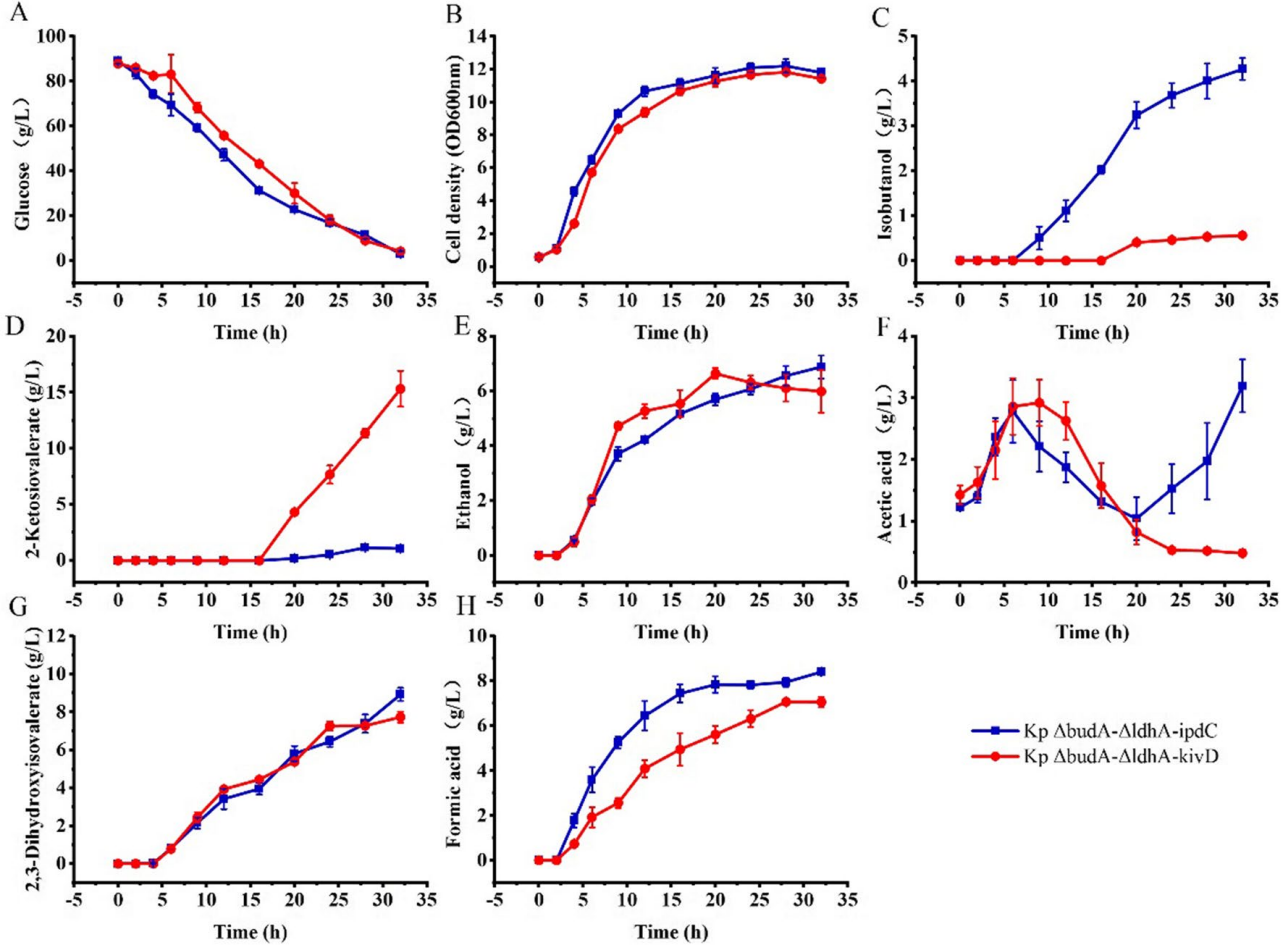
The cell growth and metabolite production of *K. pneumoniae* Δ*budA*–Δ*ldhA*–*ipdC* and *K. pneumoniae* Δ*budA*–Δ*ldhA*–*kivD* in batch culture without IPTG induction. Cells were cultured in 5 L bioreactors and no IPTG was added to the culture broth in the process. Data points are the average of *n* = 3; error bars represent standard error about the mean

To further investigate the difference of the two decarboxylases on isobutanol production, *K. pneumoniae* Δ*budA*–Δ*ldhA*–*ipdC* and *K. pneumoniae* Δ*budA*–Δ*ldhA*–*kivD* were cultured in 5 L bioreactors without induction, and fermentation results are shown in Fig. 3.

Under this condition, cell growth and metabolites production of the two strains were distinctly different. About 80 g/L of glucose was exhausted by *K. pneumoniae* Δ*budA*–Δ*ldhA*–*ipdC* after 27 h of cultivation, and the highest cell density of 12.1 OD units was achieved after 24 h. While glucose was not exhausted by *K. pneumoniae* Δ*budA*–Δ*ldhA*–*kivD* until 35 h of cultivation. Isobutanol produced by the two strains was 4.5 g/L and 0.6 g/L, respectively. In contrast to isobutanol, 2-ketoisovalerate accumulated to levels of 1.0 and 11.0 g/L for *K. pneumoniae* Δ*budA*–Δ*ldhA*–*ipdC* and *K. pneumoniae* Δ*budA*–Δ*ldhA*–*kivD*, respectively. Ethanol and acetic acid levels produced by *K. pneumoniae* Δ*budA*–Δ*ldhA*–*ipdC* were 6.8 g/L and 3.2 g/L, and they were 6.0 g/L and 0.5 g/L for *K. pneumoniae* Δ*budA*–Δ*ldhA*–*kivD*. Formate levels produced by the two strains were 8.4 and 7.1 g/L, respectively. 2,3-Dihydroxyisovalerate accumulated to levels of 9.0 and 7.7 g/L, respectively.

### Determination of kinetic parameters of Kp-IpdC and KivD

Comparing the results of batch cultures of *K. pneumoniae* Δ*budA*–Δ*ldhA*–*ipdC* and *K. pneumoniae* Δ*budA*–Δ*ldhA*–*kivD* with and without IPTG induction it can be concluded that Kp-IpdC favors isobutanol production without induction, while KivD favors isobutanol production with IPTG induction. Expression of *kivD* without induction coincided with a high level of 2-ketoisovalerate accumulation. However, expression of *ipdC* with IPTG induction did not result in a high level of 2-ketoisovalerate. Thus, expression of *ipdC* with IPTG induction might constrain the metabolic flux of 2-ketoisovalerate synthesis. To clarify this hypothesis, the kinetic parameters of the two enzymes were determined in vitro.

Pyruvate is a central metabolite of the cell and the substrate of the first reaction of the isobutanol synthesis pathway. Indole-3-pyruvate and pyruvate are both keto acids. Thus, we suspected that Kp-IpdC might be able to catalyse pyruvate decarboxylation. High levels of Kp-IpdC could lead to more pyruvate to be converted to aldehyde and further to form ethanol or acetic acid, which could limit the carbon flux of the isobutanol synthesis pathway. In vitro enzymatic reactions of 2-ketoisovalerate and pyruvate decarboxylation catalyzed by Kp-IpdC or KivD were performed. Kinetic parameters were calculated (Additional file 1: Figs. S1, S2) and results are summarized in Table 2.

**Table 2:** Kinetic parameters 2-ketoisovalerate and pyruvate decarboxylation catalyzed by Kp-IpdC or KivD

Substrate	Enzyme	*K*_m_ (mM)	*V*_max_ (mM/min)	*K*_cat_ (s^−1^)	*K*_cat_/*K*_m_ (M^−1^ s^−1^)
2-Ketoisovalerate	Kp-IpdC	1.48 (1.20, 1.75)	0.037 (0.027, 0.056)	8.05	5445.39
	KivD	4.18 (4.08, 4.23)	0.0409 (0.038, 0.044)	3.46	828.31
Pyruvate	Kp-IpdC	3.13 (2.91, 3.30)	0.0115 (0.0105, 0.0125)	0.58	183.70
	KivD	18.81 (17.15, 20.85)	0.0034 (0.0029, 0.0039)	0.28	15.09

95% asymptotic confidence interval of *K*_m_ and *V*_max_ were given in brackets

The *K*_m_ of Kp-IpdC for 2-ketoisovalerate was 1.8 mM, while that of KivD was 4.18 mM. This indicated the affinity of Kp-IpdC for 2-ketoisovalerate was stronger than that of KivD. Furthermore, the *K*_cat_ value of Kp-IpdC for 2-ketoisovalerate was higher than that of KivD. This shows Kp-IpdC is more efficient than KivD in catalysing the decarboxylation of 2-ketoisovalerate to isobutyraldehyde. Moreover, the *K*_cat_/*K*_m_ of Kp-IpdC for 2-ketoisovalerate was 5445.39 M^−1^ s^−1^, which was more than 6 times that of KivD. Thus, Kp-IpdC is preferred in catalysis the 2-ketoisovalerate decarboxylation reaction compared to KivD.

The results show that both Kp-IpdC and KivD can use pyruvate as the substrate. The *K*_m_ of Kp-IpdC for pyruvate was lower than that of KivD. The *K*_cat_ of Kp-IpdC for pyruvate was higher than that of KivD. Accordingly, *K*_cat_/*K*_m_ of Kp-IpdC for pyruvate was about 12 times higher than that of KivD. Therefore, Kp-IpdC is more efficient in catalysis of the pyruvate decarboxylation reaction than KivD showing the same behavior as with 2-ketoisovalerate as the substrate.

Based on this kinetic analysis, Kp-IpdC is favored over KivD in catalysis of 2-ketoisovalerate decarboxylation and further conversion to isobutanol. However, Kp-IpdC exhibits promiscuous pyruvate decarboxylase activity, which adversely consumes the available pyruvate precursor for isobutanol synthesis. To overcome this disadvantage, an enzyme engineering approach was used to improve the performance of Kp-IpdC.

### Homology modeling of Kp-IpdC

From the NCBI database IpdC from *Enterobacter cloacae* (Ec-IpdC—the highest sequence identity of a known structure to Kp-IpdC) was found. Ec-IpdC has a sequence identity of 62.12% with Kp-IpdC and is one residue shorter in sequence length. The crystal structure of Ec-IpdC has been determined at 2.65 Å resolution (PDB ID 1OVM). The crystal structures of Ec-IpdC and other 9 proteins were used as templates to predict the structure of Kp-IpdC.

**Fig. 4:**
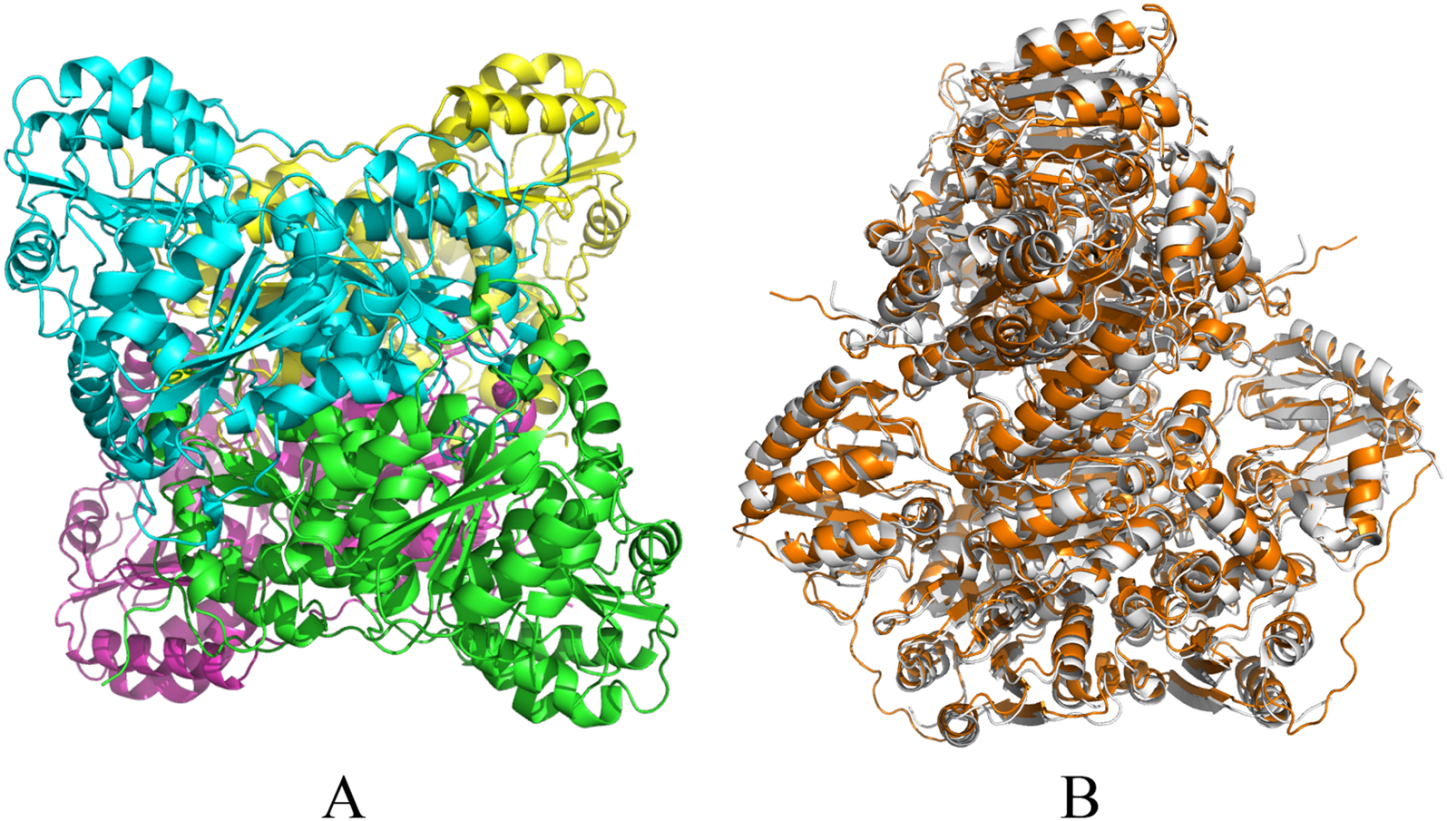
**A** The predicted tetrameric structure of Kp-IpdC; **B** Structural overlay of Kp-IpdC (orange) with Ec-IpdC (gray). The root mean square deviation (RMSD) of Kp-IpdC and Ec-IpdC monomers was 1.158

The 3D structure of Kp-IpdC predicted by RosettaCM with a structural comparison to Ec-IpdC are shown in Fig. 4.

The predicted Kp-IpdC structure was a homo-tetramer. Two monomers interact tightly to form the dimer, and two dimers form a tetramer. Each monomer consists of three domains with an open α/β class topology: the N-terminal Pyr domain (residues 3–180), which binds the pyrimidine part of ThDP; the middle domain (residues 181–340); and the C-terminal PP domain (residues 356–551), which binds the diphosphate moiety of the cofactor per subunit. The Pyr and PP domains contain a six-stranded parallel β-sheet flanked by a number of helices, whereas the middle domain contains a six-stranded mixed β-sheet, with several helices packing against the sheet.

The Root Mean Square Deviation (RMSD) of Kp-IpdC and Ec-IpdC monomers was 1.158 by calculation of Schrodinger’s protein structure alignment. Thus, the structures of Kp-IpdC and Ec-IpdC are very similar.

### Computational design of Kp-IpdC

In total 10 variants of Kp-IpdC where one or two residues were substituted with other hydrophobic amino acids were obtained through design simulation, with the assumption that altering the hydrophobic interaction at the active site might affect the substrate selectivity and preference. These variants all had favorable Rosetta energy with 2-ketoisovalerate as substrate while showing unfavorable Rosetta energy with pyruvate as substrate in docking simulations.

Genes encoding these variants of Kp-IpdC were constructed by site-directed mutagenesis and overexpressed in *E. coli*. Enzymes were purified from the lysate of these *E. coli* strains. Kinetic parameters of these enzymes with 2-ketoisovalerate or pyruvate as substrates were determined, and results are shown in Additional file 1: Figs. S3–S10 and summarized in Table 3.

**Table 3:** Kinetic parameters of variants of Kp-IpdC with 2-ketoisovalerate or pyruvate as substrates

Variants	Substrate	*K*_m_ (mM)	*V*_max_ (mM/min)	*K*_cat_ (s^−1^)	*K*_cat_/*K*_m_ (M^−1^ s^−1^)
Kp-IpdC	2-Ketoisovalerate	1.48 (1.20, 1.75)	0.037 (0.027, 0.056)	8.05	5445.39
	Pyruvate	3.13 (2.91, 3.30)	0.0115 (0.0105, 0.0125)	0.58	183.70
A387L	2-Ketoisovalerate	5.91 (3.69, 7.69)	0.0024 (0.0013, 0.0089)	0.31	52.57
	Pyruvate	13.19 (10.14, 16.38)	0.0024 (0.0020, 0.0031)	0.21	15.63
F388W	2-Ketoisovalerate	15.85 (15.22, 16.49)	0.019 (0.015, 0.024)	2.9	172.68
	Pyruvate	n.d	n.d	n.d	n.d
V542I	2-Ketoisovalerate	1.99 (1.57, 2.40)	0.018 (0.015, 0.020)	2.88	1443.98
	Pyruvate	23.38 (22.22, 24.82)	0.0056 (0.0048, 0.0067)	0.9	38.39
L546W	2-Ketoisovalerate	1.01 (0.96, 1.18)	0.034 (0.030, 0.038)	2.59	2559.61
	Pyruvate	693.2 (677.3, 888.9)	0.085 (0, 0.096)	6.66	9.61
D289L + T290L	2-Ketoisovalerate	n.d	n.d	n.d	n.d
	Pyruvate	n.d	n.d	n.d	n.d
A387I + F388W	2-Ketoisovalerate	48.07 (43.02, 52.33)	0.017 (0.006, 0.023)	0.704	14.653
	Pyruvate	n.d	n.d	n.d	n.d
D289L	2-Ketoisovalerate	n.d	n.d	n.d	n.d
	Pyruvate	n.d	n.d	n.d	n.d
T290L	2-Ketoisovalerate	0.98 (0.91, 1.05)	0.038 (0.036, 0.041)	2.97	2609.8
	Pyruvate	13.01 (9.55, 16.46)	0.0057 (0.0039, 0.001)	0.46	40.5
Q383M	2-Ketoisovalerate	1.88 (0.177, 0.199)	0.030 (0.027, 0.034)	4.18	2215.2
	Pyruvate	19.7 (18.1, 20.5)	0.016 (0.012, 0.020)	2.16	109.4
A387I	2-Ketoisovalerate	23.4 (22.9, 23.9)	0.034 (0.029, 0.041)	1.61	68.62
	Pyruvate	39.7 (36.7, 43.3)	0.0051 (0.0036, 0.009)	0.24	6.02

95% asymptotic confidence interval of *K*_m_ and *V*_max_ were given in brackets

*nd* not detectable

The D289L and D289L + T290L variants lost all decarboxylation activity with 2-ketoisovalerate and pyruvate, respectively. Whereas F388W lost all activity to catalyze pyruvate decarboxylation and was observed to have lower decarboxylation activity with 2-ketoisovalerate. The *K*_m_ and *K*_cat_/*K*_m_ of F388W for 2-ketoisovalerate were 15.85 mM and 172.68 M^−1^ s^−1,^ respectively. While those of the wild type Kp-IpdC were 1.48 mM and 5445.39 M^−1^ s^−1^ (shown in Table 2). Thus, F388W was not a suitable enzyme to be used for isobutanol production.

The *K*_m_ of A387L, V542I, A387I + F388W, Q383M, and A387L for pyruvate were all higher than that of the wild-type Kp-IpdC (3.13 mM, Table 2). However, the *K*_m_ values of these enzymes for 2-ketoisovalerate were also higher than that of the wild-type Kp-IpdC (1.48 mM, Table 2). These enzymes were all eliminated for further investigations.

Variants L546W and T290L showed lower *K*_m_ values for 2-ketoisovalerate, 1.01 and 0.98 mM, respectively, which is lower than that of the wild Kp-IpdC. The *K*_m_ of these two variants with pyruvate were 693.2 mM and 13.01 mM, respectively. These values were much higher compared to the 3.13 mM of the wild type Kp-IpdC (Table 2). The *K*_cat_/*K*_m_ values of T290L and L546W with pyruvate were 40.5 M^−1^ s^−1^ and 9.61 M^−1^ s^−1^, respectively. These values were much lower than the value of 183.70 M^−1^ s^−1^ of the wild type Kp-IpdC (Table 2).

**Fig. 5.**
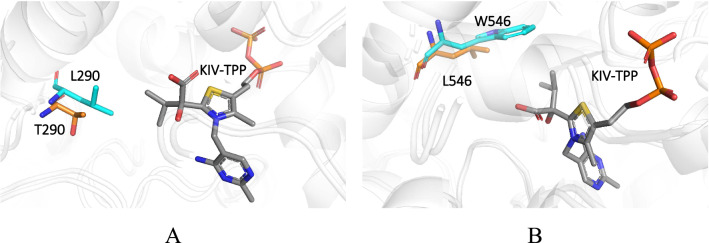
3D structure of the active site of Kp-IpdC with substrate bound obtained from simulations using PyMOL. **A** T290L. **B** L546W

The modelled 3D structures of the active site of L546W and T290L docked with 2-ketoisovalerate are shown in Fig. 5. The native substrate of Kp-IpdC is indole-3-pyruvate and its catalytic pocket is suitable for the native substrate. The molecule size of indole-3-pyruvate is larger than that of 2-ketoisovalerate. Thus, we reasoned that reducing the size of the catalytic pocket would favor 2-ketoisovalerate as a substrate. The threonine at residue 290 was mutated to leucine in T290L. The side chain of leucine is larger and more hydrophobic than that of threonine. This structure had a smaller catalytic pocket and could be more suitable for 2-ketoisovalerate–ThDP–Mg^2+^ to be bound. The molecule size of pyruvate is smaller than that of 2-ketoisovalerate, thus the catalytic pocket of T290L might not be suitable for the pyruvate–ThDP–Mg^2+^ complex. The leucine at residue 546 was mutated to tryptophan in L546W. The side chain of tryptophan is closer to 2-ketoisovalerate than that of leucine in the catalytic pocket. This could have made the 2-ketoisovalerate–ThDP–Mg^2+^ complex more stable in the catalytic pocket. The variants L546W and T290L have the characteristics of enhanced affinity interaction with 2-ketoisovalerate and reduced affinity with pyruvate. Thus, these two variants were selected to be used for isobutanol production.

### Isobutanol production using strains with variants of Kp-IpdC

**Fig. 6:**
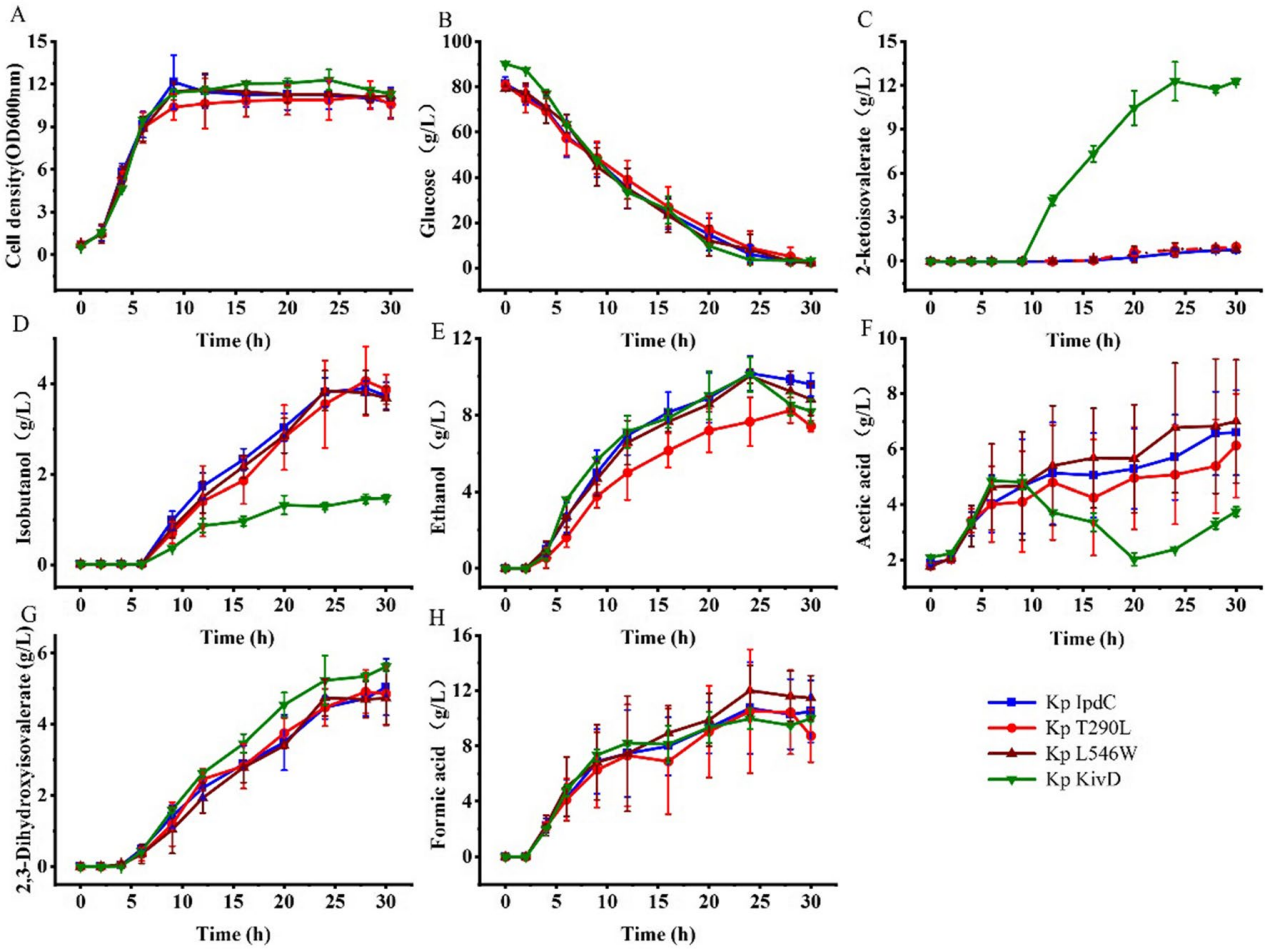
The cell growth and metabolites production of *K. pneumoniae* IpdC, *K. pneumoniae* T290L, *K. pneumoniae* L546W, and *K. pneumoniae* KivD in batch cultures with IPTG induction. Cells were cultured in 5 L bioreactors and 1 mM of IPTG was added to the culture broth after 8 h of cultivation. Data points are the average of *n* = 3; error bars represent standard error about the mean

*Klebsiella pneumoniae* Δ*budA*–Δ*ldhA*–Δ*ipdC* was constructed to eliminate the activity of endogenous Kp-IpdC. T290L and L546W were ligated to *K. pneumoniae* expression vector pDK6 and transformed into *K. pneumoniae* Δ*budA*–Δ*ldhA*–Δ*ipdC* to construct *K. pneumoniae* Δ*budA*–Δ*ldhA*–Δ*ipdC*–T290L (T290L) and *K. pneumoniae* Δ*budA*–Δ*ldhA*–Δ*ipdC*–L546W (L546W), respectively. *K. pneumoniae* Δ*budA*–Δ*ldhA*–Δ*ipdC*–*ipdC* (IpdC) and *K. pneumoniae* Δ*budA*–Δ*ldhA*–Δ*ipdC*–*kivD* (KivD) were constructed as control strains. These four strains were cultured in 5 L bioreactors with IPTG induction, and results are shown in Fig. 6.

Cell growth and glucose consumption of these strains were similar. After 30 h of cultivation, about 80 g/L of glucose was utilised completely by these strains. Similar to *K. pneumoniae* Δ*budA*–Δ*ldhA*–*kivD*, 12.3 g/L of 2-ketoisovalerate was accumulated in the broth of *K. pneumoniae* KivD after 30 h of cultivation. However, 2-ketoisovalerate levels were less than 1 g/L for the other three strains. Isobutanol produced by *K. pneumoniae* KivD was 1.5 g/L, which was distinctly lower than that of the other strains.

Specifically, 3.9 g/L, 4.1 g/L, and 3.8 g/L of isobutanol were produced by *K. pneumoniae* IpdC, *K. pneumoniae* T290L, and *K. pneumoniae* L546W, respectively, after 28 h of cultivation. Isobutanol produced by *K. pneumoniae* IpdC and other strains shown statistically significant differences (*t* test, *P* < 0.05). In addition, 10.2 g/L, 7.7 g/L, and 10.1 g/L of ethanol were produced by these strains, respectively. The decrease in isobutanol and ethanol levels towards the end of the cultivation is probably due to the evaporation that exceeds their production. All strains produced 2,3-dihydroxyisovalerate (8.7 g/L, 8.7 g/L and 8.4 g/L) and formate (8.5 g/L, 7.3 g/L and 9.8 g/L) as by-products in similar amounts.

Ethanol (7.7 g/L) and acetic acid (5.1 g/L) produced by *K. pneumoniae* T290L were reduced 24% and 7%, respectively, compared with that of *K. pneumoniae* IpdC. This indicated the decarboxylation reaction of pyruvate was reduced in *K. pneumoniae* T290L. However, there was little increase in isobutanol production by *K. pneumoniae* T290L compared to *K. pneumoniae* IpdC.

**Fig. 7:**
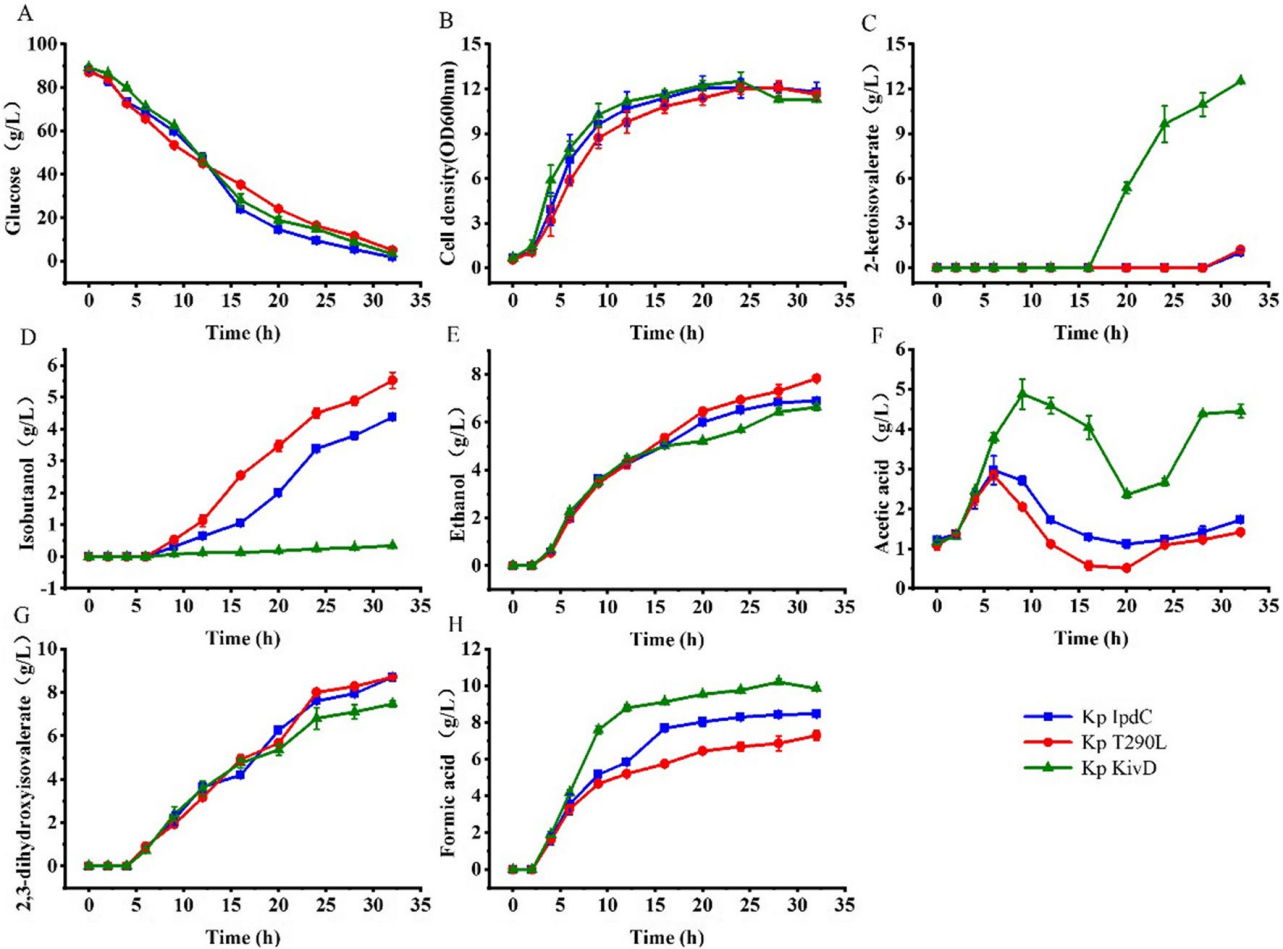
The cell growth and metabolites production of *K. pneumoniae* IpdC, *K. pneumoniae* T290L, and *K. pneumoniae* KivD in batch cultures without IPTG induction. Cells were cultured in 5 L bioreactors and no IPTG was added to the culture broth in the process. Data points are the average of *n* = 3; error bars represent standard error about the mean

Based on these results *K. pneumoniae* T290L was selected for further investigation. This strain, *K. pneumoniae* KivD and *K. pneumoniae* IpdC were cultured without IPTG induction, and the results are shown in Fig. 7.

Cell growth and glucose consumption of *K. pneumoniae* T290L were slower than that of the other two strains. About 5 g/L of glucose was unused after 32 h of cultivation. While all the glucose was consumed by the other two strains, similar to the cultivations with IPTG induction.

A high level of 2-ketoisovalerate was accumulated in the broth of *K. pneumoniae* KivD after 32 h of cultivation with a titer of 12.5 g/L. This was close to that obtained with IPTG induction. However, isobutanol produced by this strain was only 0.32 g/L compared to 1.5 g/L with induction. Acetic acid, and formate levels produced by *K. pneumoniae* KivD were higher than that of the other two strains, with the titers of 4.5 g/L and 9.8 g/L, respectively.

Isobutanol produced by *K. pneumoniae* IpdC and *K. pneumoniae* T290L were 4.4 and 5.5 g/L, respectively. These titers were both higher than that obtained with IPTG induction. Ethanol produced by *K. pneumoniae* IpdC was 6.9 g/L, which was lower than that obtained with IPTG induction. Whereas the ethanol titer produced by *K. pneumoniae* T290L was 7.8 g/L, which was nearly the same as that with IPTG induction. Acetic acid produced by the two strains was reused by the cells, with final titers of 1.7 g/L and 1.4 g/L. All final acetic acid levels were lower than that obtained with IPTG induction. In addition, both strains produced 8.7 g/L of 2,3-dihydroxyisovalerate and 8.5 g/L and 7.3 g/L of formate were produced by *K. pneumoniae* IpdC and *K. pneumoniae* T290L, respectively.

Isobutanol production by *K. pneumoniae* T290L was improved by 24% compared to that by *K. pneumoniae* IpdC without induction. However, more ethanol was produced by *K. pneumoniae* T290L in comparison to *K. pneumoniae* IpdC. By contrast, acetic acid and formate levels produced by *K. pneumoniae* T290L were decreased compared with *K. pneumoniae* IpdC. The substrate conversion ratio of glucose to isobutanol obtained by *K. pneumoniae* T290L was 6.7% (w/w) or 0.16 mol/mol. Isobutanol production by *K. pneumoniae* IpdC with IPTG induction was lower than that obtained without IPTG induction. *K. pneumoniae* T290L cultures showed a similar tendency indicating that IPTG induced expression of T290L still lead to higher pyruvate flux into by-products.

## Discussion

### Kp-IpdC is more efficient than KivD in catalysing 2-ketoisovalerate decarboxylation

A critical enzyme in the artificial isobutanol synthesis pathway is 2-keto acid decarboxylase [9], this enzyme is common in plants, yeasts, and fungi but less so in bacteria [26]. Kp-IpdC had been identified to catalyse the 2-ketoisovalerate decarboxylation reaction in *K. pneumoniae*. While all artificial isobutanol synthesis pathways using KivD from *L. lactis* to catalyse this decarboxylation reaction [9, 27–30]. Initially Kp-IpdC and KivD were both expressed in *K. pneumoniae* Δ*budA*–Δ*ldhA* without induction of IPTG. Higher level of isobutanol was obtained by *K. pneumoniae* Δ*budA*–Δ*ldhA*–*ipdC* than that of *K. pneumoniae* Δ*budA*–Δ*ldhA*–*kivD* (Fig. 3) consistent with the in vitro experimental results shown in Table 2 indicating that the efficiency of Kp-IpdC was higher than that of KivD in catalysis of 2-ketoisovalerate decarboxylation. The comparison of isobutanol production by *K. pneumoniae* KivD and *K. pneumoniae* IpdC (Figs. 6, 7) confirmed these results. Thus, Kp-IpdC is more efficient than KivD in catalysis of 2-ketoisovalerate decarboxylation. If KivD was replaced by Kp-IpdC in the artificial isobutanol synthesis pathways, the isobutanol titers could potentially be significantly improved.

### Catalysis of pyruvate decarboxylation is a limitation of Kp-IpdC

pDK6 is a multicopy vector used for protein expression in *K. pneumoniae* and this plasmid uses the tac promoter for gene expression. With IPTG induction, the protein expressed would constitute more than 1% of total cellular protein. In the absence of induction, the protein was also expressed to a certain level [19]. In the no-induction fermentations, low levels of 2-ketoisovalerate were accumulated in the culture broth of *K. pneumoniae* Δ*budA*–Δ*ldhA*–*ipdC* (Fig. 3). This indicated the decarboxylation reaction was still a limiting step of the isobutanol synthesis pathway. However, only a low level of isobutanol was obtained in the IPTG induced culture of *K. pneumoniae* Δ*budA*–Δ*ldhA*–*ipdC*. Furthermore, the 2-ketoisovalerate accumulated in the culture broth of *K. pneumoniae* Δ*budA*–Δ*ldhA*–*ipdC* was lower than that of *K. pneumoniae* Δ*budA*–Δ*ldhA*–*kivD*. We can conclude that the total carbon flux of the isobutanol synthesis pathway was more reduced in the IPTG induction conditions compared to the no-induction condition. High levels of ethanol and acetic acid were obtained in the culture broth of *K. pneumoniae* Δ*budA*–Δ*ldhA*–*ipdC* with IPTG induction, this indicated more pyruvate was converted to ethanol or acetic acid, instead of being used for isobutanol synthesis.

Kp-IpdC and KivD are both ThDP-dependent decarboxylases. However, the substrate range of decarboxylases can be different with some classes, such as pyruvate decarboxylases, benzoylformate decarboxylases and benzaldehyde lyases from bacteria or yeast accepting a broad variety of substrates, including keto acids and aldehydes [31]. The substrates of Ec-IpdC are limited to keto acids. The enzyme has the highest catalytic efficiency to the native substrate indole pyruvate (*K*_m_ = 20 μM), to 4-Cl-benzoylformate and to 4-Br-benzoylformate. Pyruvate is also a substrate of this enzyme, but it has a very low affinity (*K*_m_ = 3.38 mM) [32]. These data agree with the results obtained in this study, i.e., the *K*_m_ of Kp-IpdC to pyruvate was found to be 3.31 mM (Table 2).

Pyruvate conversion by Kp-IpdC is a disadvantage for isobutanol production. A high level of Kp-IpdC leads to more pyruvate being decarboxylated to aldehyde and reduces the available pyruvate for isobutanol synthesis. This could explain the low titer of isobutanol found when *ipdC* was induced (Fig. 1).

### Protein engineering to improve the substrate specificity of Kp-IpdC

Indole pyruvate decarboxylases use thiamine diphosphate (ThDP) as a cofactor and require magnesium ions for catalytic activity. ThDP dependent enzymes catalyse a broad range of different reactions involving cleavage and formation of C–C bonds, which are essential in many biosynthetic pathways [31]. The decarboxylase superfamily contains more than ten families of decarboxylases, and their structures are highly conserved [33]. The structures comprise three similarly sized domains: the N-terminal domain which binds the pyrimidine (Pyr) ring of ThDP, a middle domain and the C-terminal domain which binds the diphosphate (PP) moiety. The active site is located at the interface between two monomers, with ThDP interacting with the Pyr domain of one monomer and the PP domain of the second [34].

Previously, different variants of decarboxylases have been constructed to alter their substrate specificity. For example, I472A of pyruvate decarboxylase (PDC) from *Zymomonas mobilis* enlarges the substrate binding site and allows the decarboxylation of longer aliphatic 2-keto acids (C4–C6) as well as aromatic 2-keto acids besides pyruvate [35]. Benzoylformate decarboxylase (BFD) from *Pseudomonas putida* favours aromatic 2-keto acids as substrate. BFD A460I can use pyruvate as the substrate, while the wild type of BFD is unable to convert it [36]. KivD was modified to change its substrate specificity. A F381L/V461A variant had a higher selectivity toward 2-keto-4-methylhexanoate [37]. A G402V/M538L/F542V variant showed a 600-fold improvement in specificity for C8 compared to C5 substrates [38]. A V461I/S286T variant had a 2.4 times improvement of the final product ratio of isobutanol to 3-methyl-1-butanol [39]. These successful examples demonstrate the application potential of decarboxylases and feasibility of changing their substrate specificities by point mutations.

Though many protein engineering works have been done, the activities of variants with non-native substrates were still much lower than that using native substrates. *K*_m_ of T290L and L546W of Kp-IpdC to 2-ketoisovalerate determined in this study were 0.98 mM and 1.01 mM. These values are both higher than the *K*_m_ of Ec-IpdC to its native substrate indole pyruvate (*K*_m_ 20 μM) [32]. Although isobutanol production by *K. pneumoniae* T290L was improved compared with *K. pneumoniae* IpdC, isobutanol production by this strain with IPTG induction was still lower than that without IPTG induction, like that of *K. pneumoniae* IpdC. The pyruvate decarboxylation activity of T290L still affects isobutanol synthesis, and this unwanted activity was not erased totally. Thus, there is still a large potential to improve the performance of Kp-IpdC.

## Conclusions

A maximum of 5.5 g/L of isobutanol was produced by *K. pneumoniae* T290L in batch culture with a substrate conversion ratio of 0.16 mol/mol, which was 25% higher than that of the control strain. However, several by-products of this strain still exhibited high levels and the isobutanol production is constrained by undesirable enzyme promiscuity of Kp-IpdC towards pyruvate. The protein engineering work showed promising results but there is scope for further improvement. One target could be to reduce the *K*_m_ of Kp-IpdC to 2-ketoisovalerate to around 20 μM, near that of Ec-IpdC to indole pyruvate, to increase the efficiency of the biological route of isobutanol production further.

## Supplementary Information

**Additional file 1: Table S1.** Oligonucleotides used for PCR. **Fig. S1.** IpdC kinetic parameters determination using Lineweaver–Burk plots. **Fig. S2.** KivD kinetic parameters determination using Lineweaver–Burk plots. **Fig. S3.** A378L kinetic parameters determination using Lineweaver–Burk plots. **Fig. S4.** F388W kinetic parameters determination using Lineweaver–Burk plots. **Fig. S5.** V541I kinetic parameters determination using Lineweaver–Burk plots. **Fig. S6.** L546W kinetic parameters determination using Lineweaver–Burk plots. **Fig. S7.** A387I + F388W kinetic parameters determination using Lineweaver–Burk plots. **Fig. S8.** T290L kinetic parameters determination using Lineweaver–Burk plots. **Fig. S9.** Q383M kinetic parameters determination using Lineweaver–Burk plots. **Fig. S10.** A387I kinetic parameters determination using Lineweaver–Burk plots.

## Data Availability

The authors declare that the data supporting the findings of this study are available within the article and its additional information files.
