# Supplementary material for: The pyruvate decarboxylase activity of IpdC is a limitation for isobutanol production by Klebsiella pneumoniae
Source: Biotechnol Biofuels Bioprod. 2022 May 2;15:41. doi: 10.1186/s13068-022-02144-8 (PMC9063327; doi:10.1186/s13068-022-02144-8)

The pyruvate decarboxylase activity of IpdC is a limitation for isobutanol production by *Klebsiella pneumoniae*

Lin Shu^1,4^#, Jinjie Gu^1,3,4^#, Qinghui Wang^1^, Shaoqi Sun^1^, Youtian Cui^3^, Jason Fell^3^, Wai Shun Mak^3^, Justin B. Siegel^3^, Jiping Shi^1^, Gary J. Lye^2^, Frank Baganz^2^*, Jian Hao^1,2,4^*

1. Lab of Biorefinery, Shanghai Advanced Research Institute, Chinese Academy of Sciences, No. 99 Haike Road, Pudong, Shanghai, 201210, PR China

2. Department of Biochemical Engineering, University College London, Gordon Street, London WC1H 0AH, UK

3. Department of Chemistry, Biochemistry & Molecular Medicine, and the Genome Center, University of California, Davis, One Shields Avenue, Davis, California 95616, USA

4. University of Chinese Academy of Sciences, Beijing, 100049, PR China

# Both authors contributed equally to this work

*Corresponding author.

Email: haoj@sari.ac.cn

f.baganz@ucl.ac.uk

Tel.: +86 21 20325163

Table S1 Oligonucleotides used for PCR

| Primers | Nucleotide sequence(5’-3’) |
| --- | --- |
| ipdC-s | AGCTAGAATTCATGCAACCGACCTACACTATTGGGG |
| ipdC-a | AGCTAGGATCCCTAAACGCGGCTGTTTCGTTCCT |
| kivD-s | AGCTAGGTACCATGTATACAGTAGGAGATTACCTATTAGACCG |
| kivD-a | AGCTAGGATCCTTATGATTTATTTTGTTCAGCAAATAGTTTGCCCATTTTTTTC |
| D289L-s | GGCACGCGCTTCACCCTCACCATCACCGCGGG |
| D289L-a | CCCGCGGTGATGGTGAGGGTGAAGCGCGTGCC |
| T290L-s | CGCGCTTCACCGACCTCATCACCGCGGGATTC |
| T290L-a | GAATCCCGCGGTGATGAGGTCGGTGAAGCGCG |
| Q383M-s | CGATATTATTCTCGCCGACATGGGGACGGCCG |
| Q383M-a | CGGCCGTCCCCATGTCGGCGAGAATAATATCG |
| A387I-s | CCAGGGGACGGCCATCTTCGGCATTGCCGCGC |
| A387I-a | GCGCGGCAATGCCGAAGATGGCCGTCCCCTGG |
| D289L+T290L-s | GCACGCGCTTCACCCTCCTCATCACCGCGGGATTC |
| D289L+T290L-a | GAATCCCGCGGTGATGAGGAGGGTGAAGCGCGTGC |
| F388W-s | CCAGGGGACGGCCGCCTGGGGCATTGCCGCGC |
| F388W-a | GCGCGGCAATGCCCCAGGCGGCCGTCCCCTGG |
| A387L-s | CCAGGGGACGGCCCTCTTCGGCATTGCCGCGC |
| A387L-a | GCGCGGCAATGCCGAAGAGGGCCGTCCCCTGG |
| V542I-s | CCGATTTCCTGCGCGCGATCACGCAGGCGCTGGAGG |
| V542I-a | CCTCCAGCGCCTGCGTGATCGCGCGCAGGAAATCGG |
| L546W-s | GGTGACGCAGGCGTGGGAGGAACGAAACAGCCG |
| L546W-a | CGGCTGTTTCGTTCCTCCCACGCCTGCGTCACC |
| A387I+F388W-s | CCGACCAGGGGACGGCCATCTGGGGCATTGCCGCGC |
| A387I+F388W-a | GCGCGGCAATGCCCCAGATGGCCGTCCCCTGGTCGG |
|  |  |

Fig S1. IpdC kinetic parameters determination using Lineweaver-Burk plots.

Enzyme concentration 4.6 mg/L


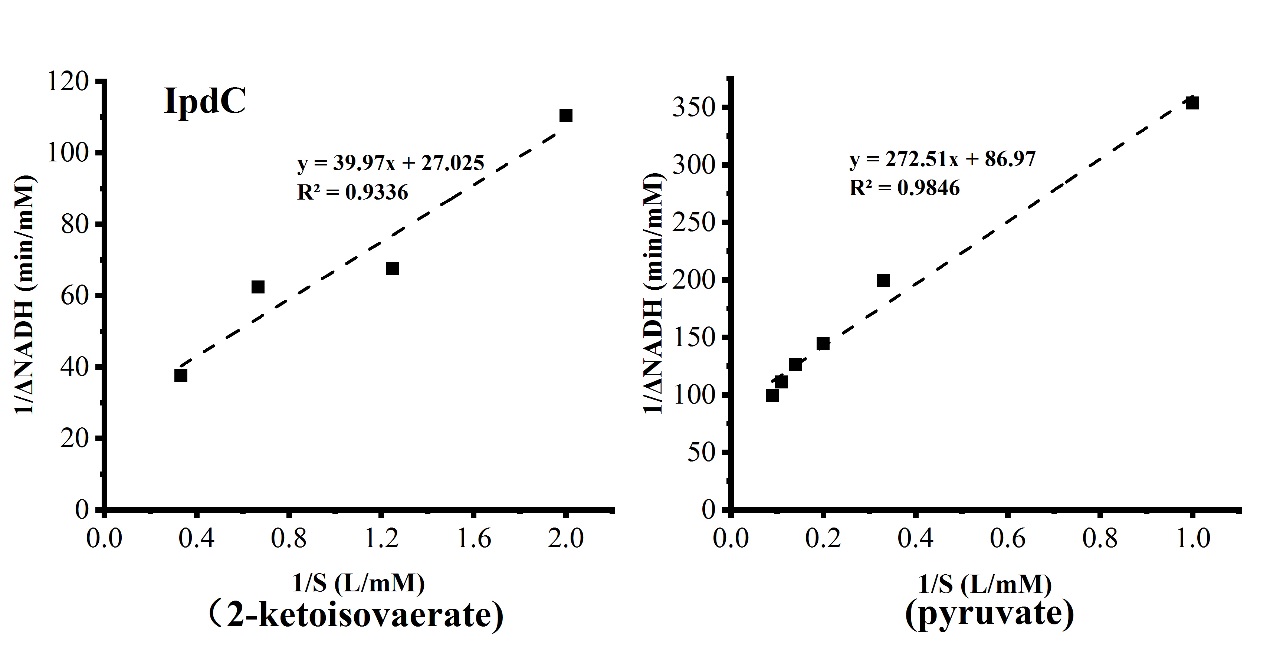


Fig S2. KivD kinetic parameters determination using Lineweaver-Burk plots.

Enzyme concentration 15.2 mg/L


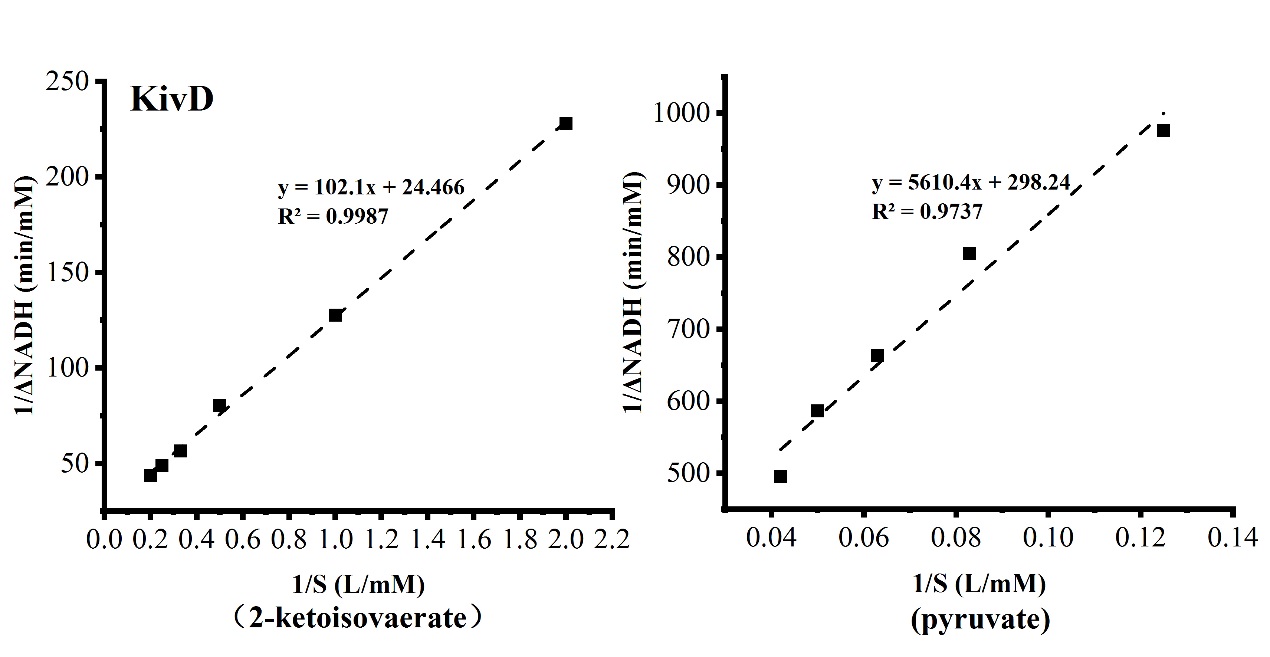


Fig S3. A378L kinetic parameters determination using Lineweaver-Burk plots.

Enzyme concentration 7.8 mg/L


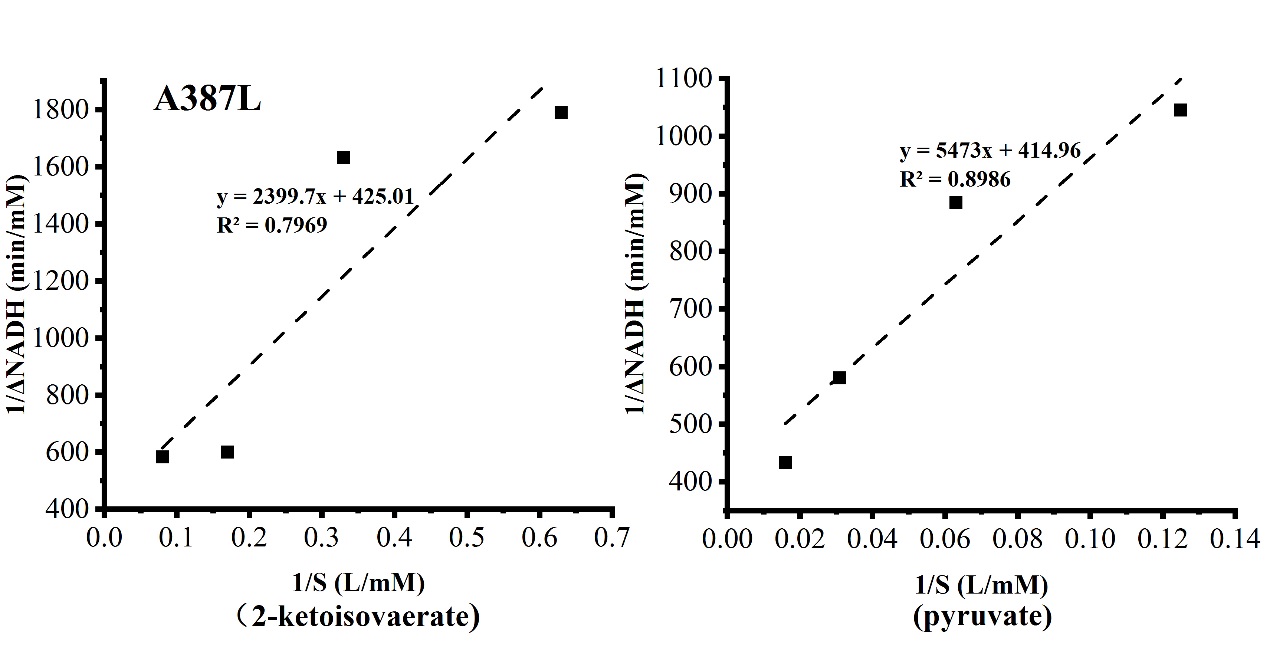


Fig S4. F388W kinetic parameters determination using Lineweaver-Burk plots.

Enzyme concentration 6.8 mg/L


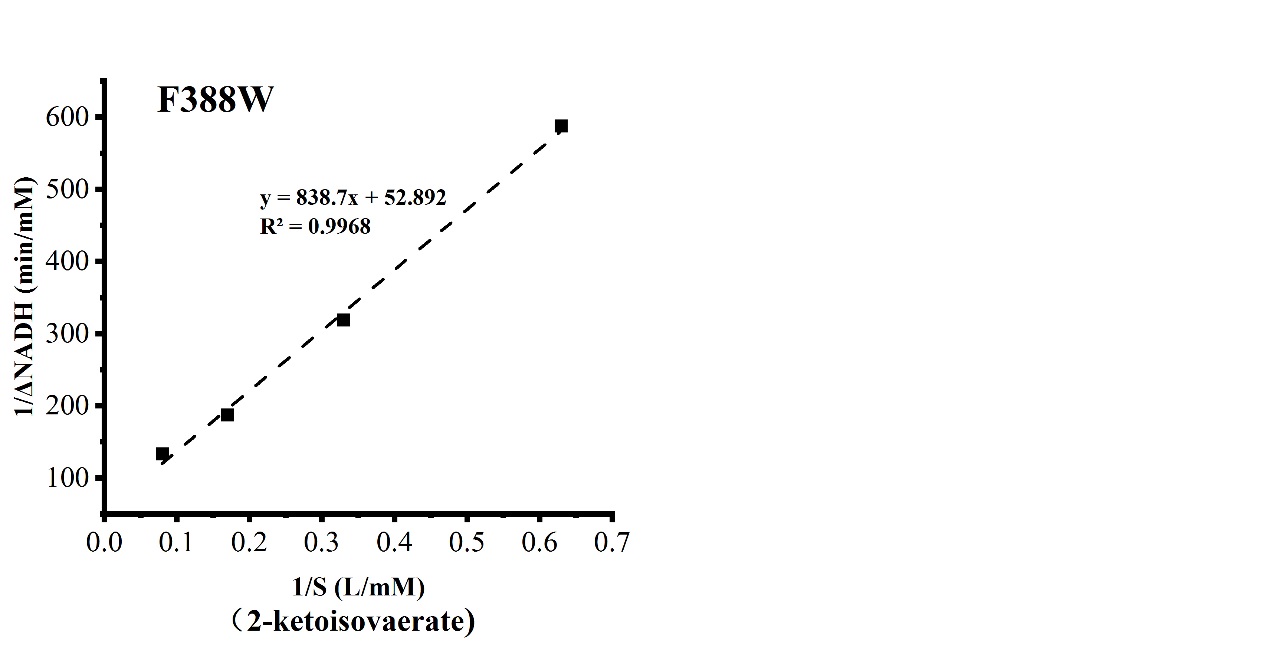


Fig S5. V541I kinetic parameters determination using Lineweaver-Burk plots.

Enzyme concentration 6.2 mg/L


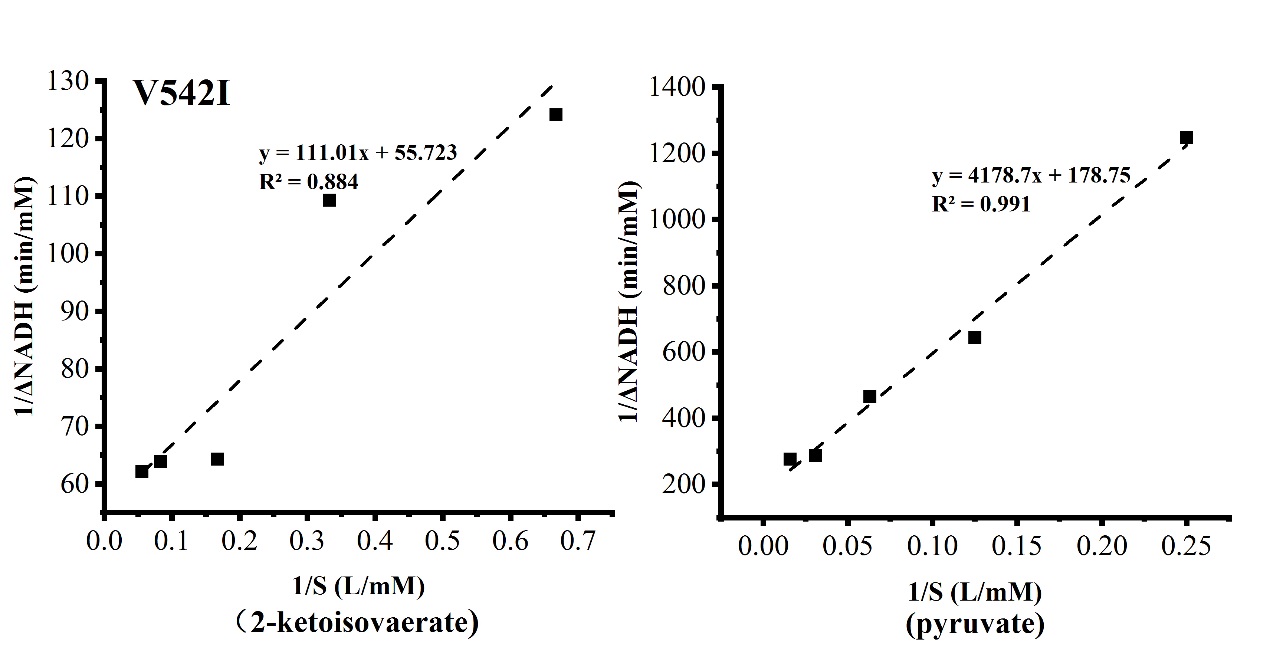


Fig S6. L546W kinetic parameters determination using Lineweaver-Burk plots.

Enzyme concentration 13.0 mg/L


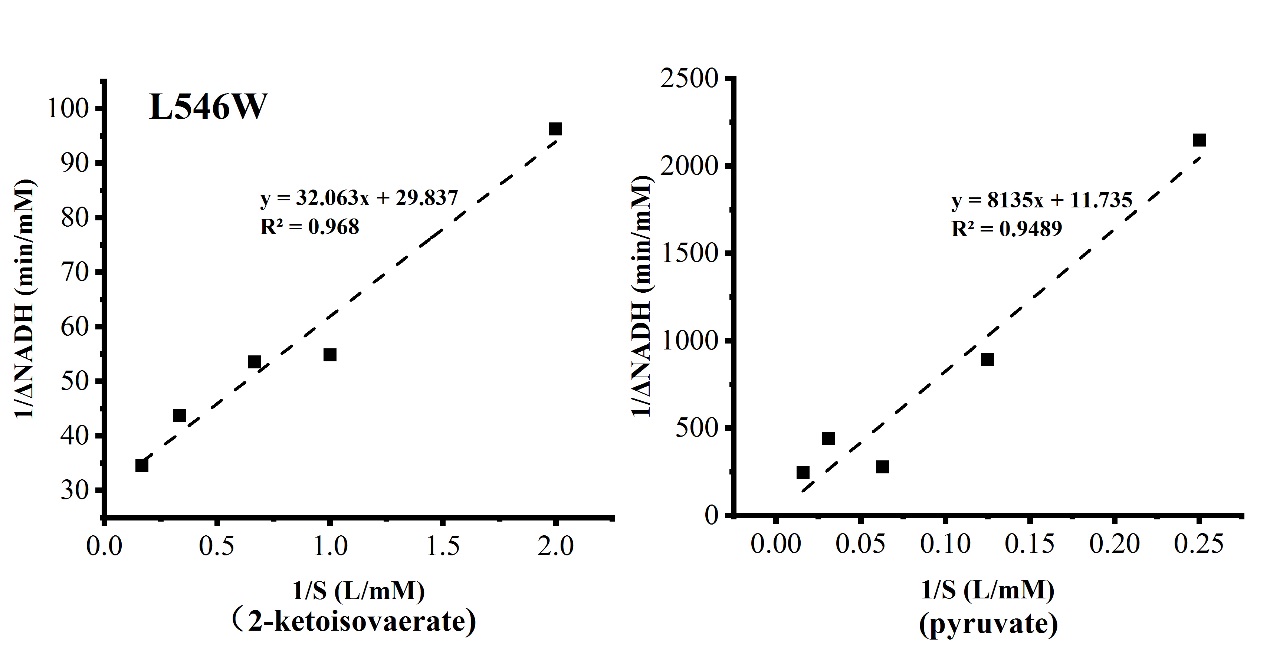


Fig S7. A387I +F388W kinetic parameters determination using Lineweaver-Burk plots.

Enzyme concentration 23.6 mg/L


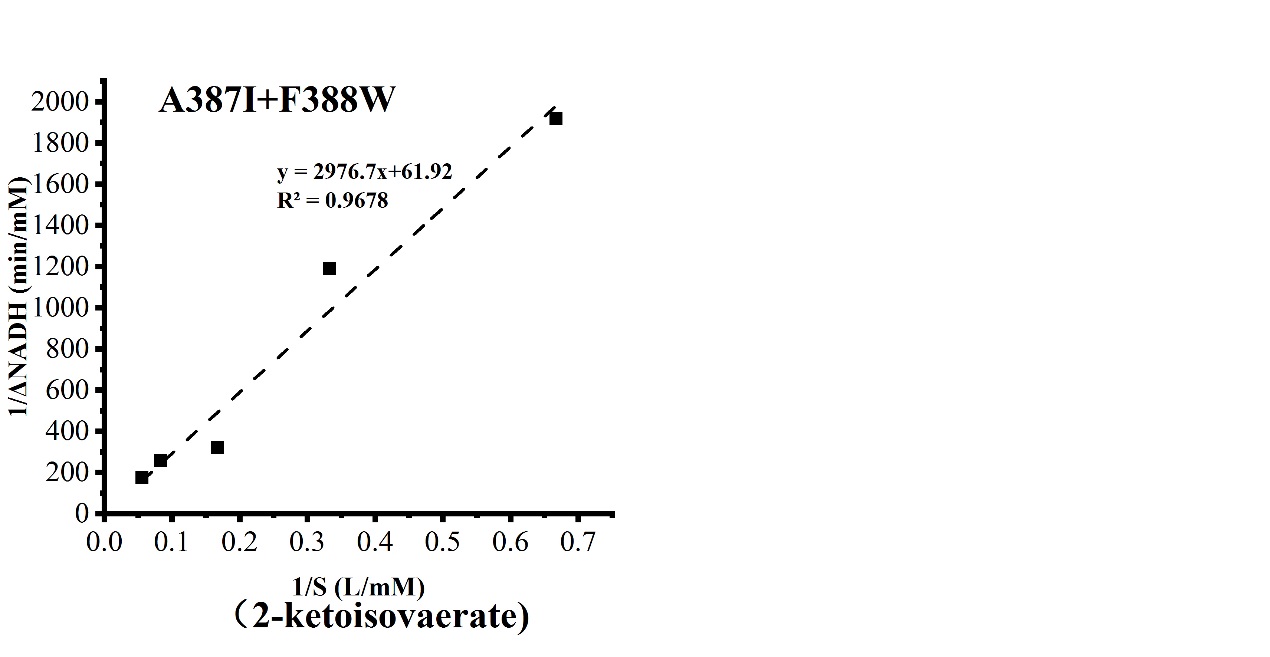


Fig S8. T290L kinetic parameters determination using Lineweaver-Burk plots.

Enzyme concentration 12.9 mg/L


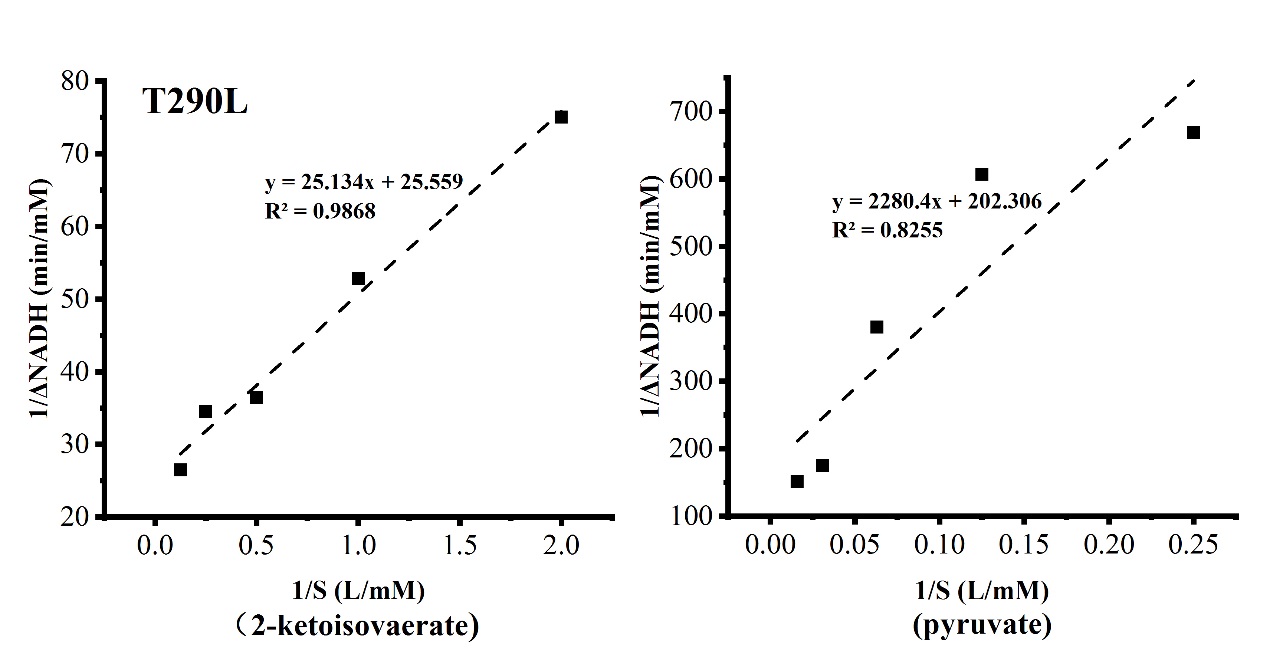


Fig S9. Q383M kinetic parameters determination using Lineweaver-Burk plots.

Enzyme concentration 7.2 mg/L


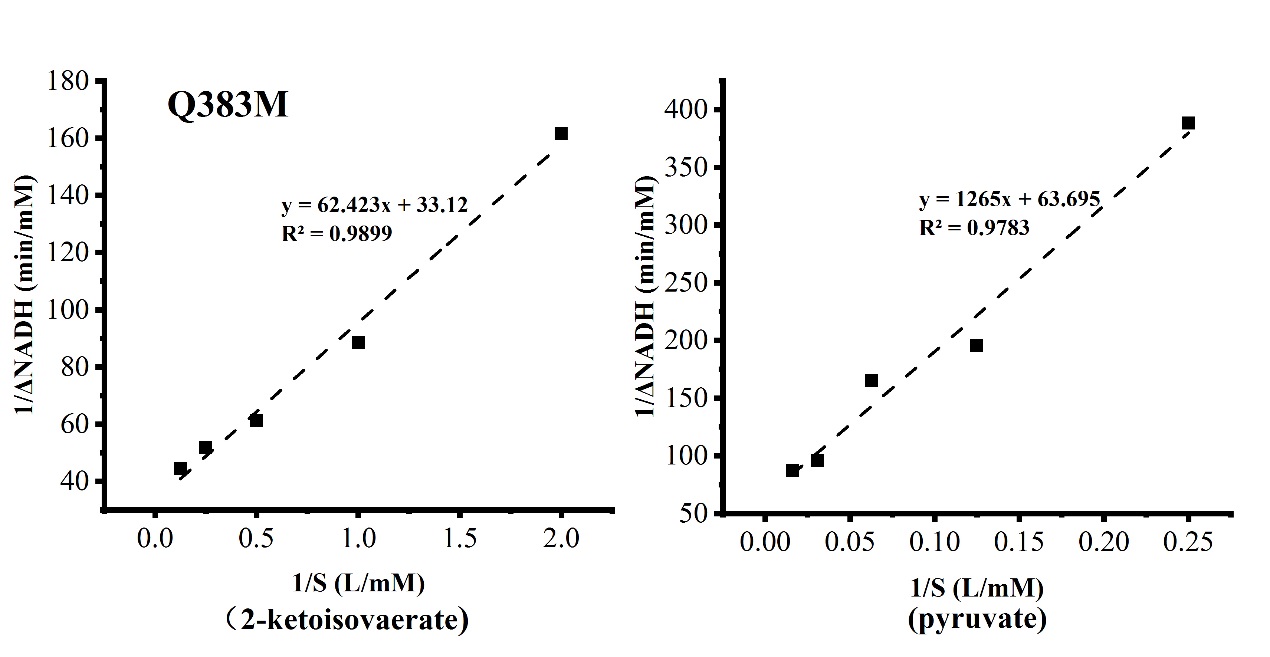


Fig S10. A387I kinetic parameters determination using Lineweaver-Burk plots.

Enzyme concentration 21.5 mg/L


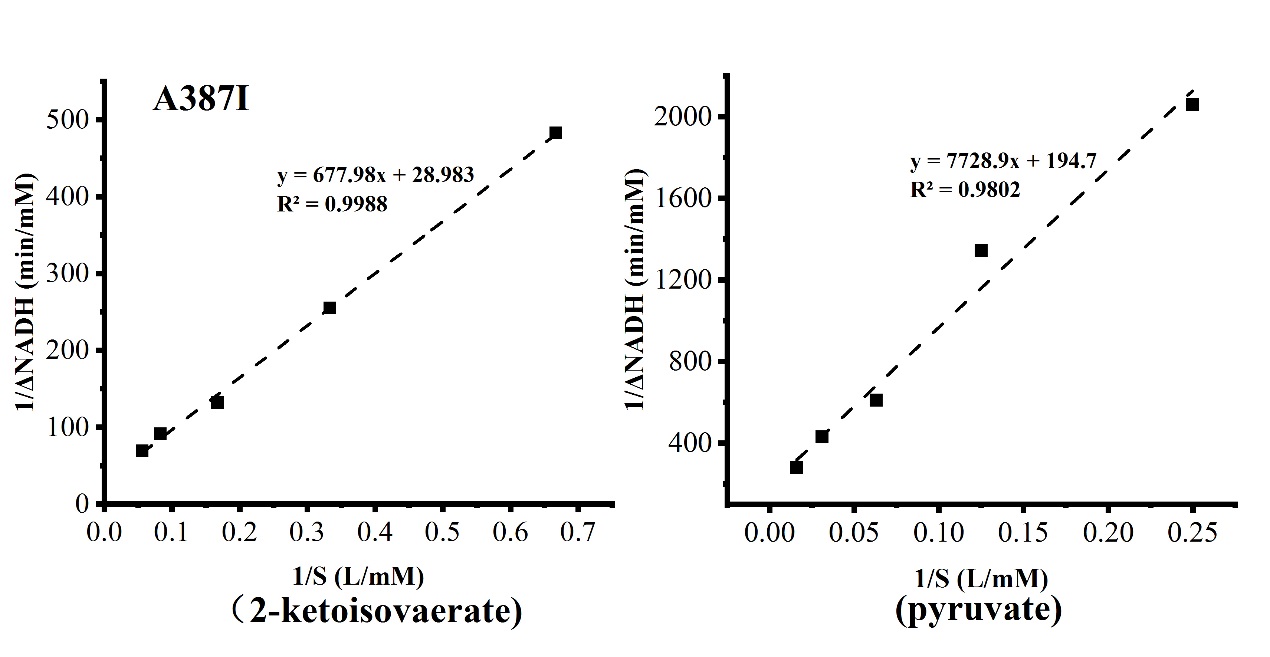

Supplement: Supplementary file 1 — Additional file 1: Table S1. Oligonucleotides used for PCR. Fig. S1. IpdC kinetic parameters determination using Lineweaver–Burk plots. Fig. S2. KivD kinetic parameters determination using Lineweaver–Burk plots. Fig. S3. A378L kinetic parameters determination using Lineweaver–Burk plots. Fig. S4. F388W kinetic parameters determination using Lineweaver–Burk plots. Fig. S5. V541I kinetic parameters determination using Lineweaver–Burk plots. Fig. S6. L546W kinetic parameters determination using Lineweaver–Burk plots. Fig. S7. A387I + F388W kinetic parameters determination using Lineweaver–Burk plots. Fig. S8. T290L kinetic parameters determination using Lineweaver–Burk plots. Fig. S9. Q383M kinetic parameters determination using Lineweaver–Burk plots. Fig. S10. A387I kinetic parameters determination using Lineweaver–Burk plots. [file 13068_2022_2144_MOESM1_ESM.docx]
